# Secondary Prevention after Minor Stroke and TIA - Usual Care and Development of a Support Program

**DOI:** 10.1371/journal.pone.0049985

**Published:** 2012-12-17

**Authors:** Stefanie Leistner, Steffen Benik, Inga Laumeier, Annerose Ziegler, Gabriele Nieweler, Christian H. Nolte, Peter U. Heuschmann, Heinrich J. Audebert

**Affiliations:** 1 Department of Neurology, Charité-Universitätsmedizin Berlin, Berlin, Germany; 2 Institute of Clinical Epidemiology and Biometry, University of Würzburg, Würzburg, Germany; 3 Department of Neurology, Center for Stroke Research, Charité-Universitätsmedizin Berlin, Berlin, Germany; S.G.Battista Hospital, Italy

## Abstract

**Background:**

Effective methods of secondary prevention after stroke or TIA are available but adherence to recommended evidence-based treatments is often poor. The study aimed to determine the quality of secondary prevention in usual care and to develop a stepwise modeled support program.

**Methods:**

Two consecutive cohorts of patients with acute minor stroke or TIA undergoing usual outpatient care versus a secondary prevention program were compared. Risk factor control and medication adherence were assessed in 6-month follow-ups (6M-FU). Usual care consisted of detailed information concerning vascular risk factor targets given at discharge and regular outpatient care by primary care physicians. The stepwise modeled support program additionally employed up to four outpatient appointments. A combination of educational and behavioral strategies was employed.

**Results:**

168 patients in the observational cohort who stated their openness to participate in a prevention program (mean age 64.7 y, admission blood pressure (BP): 155/84 mmHg) and 173 patients participating in the support program (mean age 67.6 y, BP: 161/84 mmHg) were assessed at 6 months. Proportions of patients with BP according to guidelines were 50% in usual-care and 77% in the support program (p<0.01). LDL<100 mg/dl was measured in 62 versus 71% (p = 0.12). Proportions of patients who stopped smoking were 50 versus 79% (p<0.01). 72 versus 89% of patients with atrial fibrillation were on oral anticoagulation (p = 0.09).

**Conclusions:**

Risk factor control remains unsatisfactory in usual care. Targets of secondary prevention were met more often within the supported cohort. Effects on (cerebro-)vascular recurrence rates are going to be assessed in a multicenter randomized trial.

## Introduction

In Germany, more than 50% of all acute stroke and TIA patients admitted to hospitals survive the acute in-hospital stay without relevant disability in activities of daily living [1], [2]. However, these patients carry a high risk for recurrent vascular events. Depending on etiology, the natural risk for recurrent stroke during the first year after event has been described between 12% and 20% for stroke patients with atrial fibrillation [3], 13% with high-grade carotid artery stenosis [4], 20% in intracranial stenoses [5] and 5% without these high-risk factors [6]–[8]. A secondary ischemic event has considerably more serious consequences leading to physical disability in more than 60% and death in more than 20% of patients [9]. Patients with cerebrovascular disease are also at high risk for other ischemic events such as cardiovascular death or non-fatal myocardial infarction [10].

Growing evidence suggests that early diagnostic work-up with consequent initiation of preventive measures results in a major reduction of re-events [11]. However, recommendations are often not met in outpatient health care. Although patients who experienced a recent cerebrovascular event have a much higher vascular risk compared to the age adjusted population even with the same risk factors [10], they often do not receive more intensive care than patients subject to primary prevention [12], [13].

Reasons for non-adherence to medical recommendations include patient related factors (e.g. non-complying with polypharmaceutical regimens particularly in geriatric patients, fading motivation with time from initial event) and treatment decisions by outpatient physicians [14].

While evidence is limited with respect to prevention programs after stroke, there are encouraging experiences in other diseases, e.g. diabetes mellitus and coronary heart disease: Implementing an intensified drug therapy combined with a modification of behavior significantly reduced the risk of vascular events [15], [16]. Hence, the concept of a multimodal secondary prevention program appears to be promising in patients after TIA and minor stroke but evidence for its effectiveness is still lacking.

### Aims

The aim of the present study was to determine the quality of risk factor control after TIA and minor stroke in usual care, and to develop a practical program for supported secondary prevention in preparation of a prospective, randomized intervention trial.

## Methods

### Ethics Statement

Both studies were approved by the Ethics Committee of the Charité Universitätsmedizin Berlin. The studies have been conducted according to the principles expressed in the Declaration of Helsinki.

### The study was conducted in two parts:

#### Part A

A prospective observation aiming to analyze the current situation regarding frequency of cardiovascular risk factors, lifestyle and quality of secondary prevention in consecutive patients after recent TIA or minor stroke. In order to evaluate the motivation for participation in a supported secondary prevention program, patients were asked if they were willing to participate in a future program. Quality of secondary prevention was assessed after 6 months.

#### Part B

The modeling of a supported secondary prevention program (MODUS) – was aimed to develop strategies for intensified prevention of vascular events after TIA and minor stroke, to test the practicability, optimal intensity and acceptance of measures, and to evaluate their effects on surrogate prevention targets. Patients were invited to a modeled secondary prevention program with 3 to 4 appointments over a period of 6 months.

### Observational study (Part A)

Consecutive acute patients (≤7 days) with TIA (complete symptom remission ≤24 h, without diffusion-weighted MRI lesion) or minor stroke (mRS≤2 at time of screening) admitted to the Stroke Unit Department at the Charité Campus Benjamin Franklin were included during in-hospital stay (baseline assessment) and followed-up after 6 months.

Inclusion criteria consisted of age over 18 years, written informed consent, and at least one modifiable risk factor such as arterial hypertension, diabetes mellitus, atrial fibrillation or smoking. The following exclusion criteria were applied: TIA or minor stroke of etiologies without an evidence-based option for secondary prevention such as dissection or vasculitis, physical impairment with need of assistance for physical self-care (modified Rankin-Score>2), relevant cognitive impairment (Montreal Cognitive Assessment<25), dysphasia, severe alcohol abuse, and malignant disease with life-expectancy less than one year.

Standardized baseline assessment included a questionnaire regarding demographic information, risk factors and co-morbidities, clinical symptoms of the acute cerebrovascular event, and duration of symptoms. The clinical evaluation consisted of a neurological examination according to the National Institutes of Health Stroke Scale (NIHSS) at admission and modified Rankin Scale (mRS) as well as Barthel Index (BI) at time of study inclusion. Stroke etiology was classified according to TOAST criteria (the Trial of ORG 10172 in Acute Stroke Treatment) [17]. A venous blood sample was drawn for testing LDL cholesterol [mg/dl], CRP [mg/dl], HbA1c [%], and INR. Body mass index (BMI, kg/m2) was calculated from height and weight measurements. Blood pressure was measured manually in sitting patients (resting at least 5 minutes) on both arms. Both blood pressure values were recorded and the higher value was used for statistical comparisons. Arterial hypertension was defined by either repeated elevated systolic blood pressure >140 and/or diastolic blood pressure >90 mm Hg or the previous use of antihypertensive drugs. Diabetes was defined by either HbA1c ≥6.5% or use of antidiabetics. Hyperlipidemia was defined by either LDL cholesterol >100 mg/dl or use of lipid-lowering drugs. Current smokers were evaluated in pack years. Patients who did not currently smoke were divided in non-smokers and past smokers (≥5 years). Physical activity was measured as frequency of “physical activity with an intensity leading to transpiration over more than 30 minutes” per week.

All patients received information concerning vascular risk factor targets including written recommendations at discharge and regular outpatient care by Primary Care Physicians (PCP). Patients were asked whether they would be willing to participate in a prevention support program if available.

Follow-up (FU) was done after 6 months during an outpatient appointment assessing risk factor control according to the above mentioned definitions and medication adherence. Patients were asked about vascular re-events that were defined as myocardial infarction, stroke or TIA.

### Modeling of a supported secondary prevention program (MODUS) (Part B)

Inclusion criteria, exclusion criteria and diagnostic work-up (baseline assessment) were the same as in Part A. In addition, a combination of educational and behavioral strategies was employed to improve compliance. All involved doctors and prevention assistants underwent training according to the motivational interviewing standards [18].

The content of the support program was structured in five modules according to the major prevention targets and evidence based therapeutic options. Since arterial hypertension is a major risk factor for all sorts of vascular re-events regardless of etiology, special emphasis was placed on the blood pressure control in all patients. For the positive effects on several risk factors, all patients were also included in the ‘physical activity’ module. The other intervention modules (anticoagulant/antiplatelet therapy, nicotine cessation and nutrition/metabolism counseling) were only applied to those patients, who had the specific risk factors. The findings of risk factor as well as medication assessment were recorded and sent to the patient's PCP's together with a recommendation for optimal prevention.

### Module-based support program

#### Blood Pressure module

Blood pressure control was performed according to the recommendations of the German Neurological Society (DGN) (target<140/90 mmHg and ≤130/80 in diabetic patients [19]. Detailed information about the role of arterial hypertension in pathogenesis of stroke was provided. Since paradoxical nocturnal hypertension is frequently related to obstructive sleep apnea syndrome [20], all patients were asked about their sleep patterns. 24-hour blood pressure monitoring was performed in patients who had elevated blood pressure (values >160 mmHg systolic or >100 mmHg diastolic and in cases of suspected abnormal sleep patterns.

#### Physical activity module

Using the methods of motivational interviewing, patients were encouraged to maintain a high level of physical activity in accordance with the recommendations of the DGN (target: physical activity ≥30 min/d at least 3 times per week). Patients were informed about healthy training guidelines and individually designed activity programs. The amount of physical activity was documented. Contacts with sport groups and centers promoting physical activity were facilitated.

#### Anticoagulant/antiplatelet therapy module

Depending on the etiology of stroke, the specific drug therapy was determined (oral anticoagulation with target INR 2–3 in patients with atrial fibrillation, single or combined antiplatelet therapy in patients with arterial stroke etiology). The adherence to anticoagulant/antiplatelet therapy was regularly monitored. Calendars were used to enhance compliance. Patient self-monitoring (INR measurements) was proposed to appropriate patients. INR was measured in patients with oral anticoagulation.

#### Nicotine cessation module

Smokers were encouraged and supported in their efforts to stop smoking. Group support was offered and the patient's relatives were involved in counseling sessions.

#### Nutrition and metabolism counseling module

Patients were supported by means of individual nutrition counseling. They were informed in detail on the potential benefits of the Mediterranean [21] and DASH [22], [23] diets with respect to their individual risk factors (such as diabetes, hypertension and hypercholesterolemia).

In order to determine the optimal intensity of the supported prevention program, a possible modification of the support program was planed a priori. After 5 months and 10 months, two interim analyses were performed. Since risk factor targets were not satisfactorily met, the intervention schemes were modified (intensified).

#### Level 1

All patients had outpatient appointments at 6 weeks, 3 months and 6 months after discharge. Patients received a summary of the assessed parameters including written recommendations. They were encouraged to forward this summary to their general practitioners (PCP).

#### Level 2

Patients had the same scheduled outpatient appointments. Additionally, the PCPs were informed directly via telephone when the risk factor assessment revealed values beyond the target range and were asked whether they would agree to immediate change of medication including prescription in the outpatient clinic.

#### Level 3

These patients additionally received baseline counseling after study inclusion but before discharge and one additional appointment three weeks after discharge. Information to the PCPs was managed according to level 2. In case of blood pressure values >160 mmHg systolic or >100 mmHg diastolic, patients were invited to a repeated 24 h blood pressure measurement within 3 weeks independent of a scheduled visit.

Assessment and documentation of risk factors was conducted the same way as in the observational cohort.

### Statistical analyses

For the nature of the presented study, all analyses are presented in a descriptive manner. Values are described as mean with standard deviation (SD) and/or median with interquartile ranges (IQR). Statistical calculations were performed with SPSS-18® software.

### Standard Protocol Approvals and Patient Consents

The dataflow was approved by institutional representatives for data protection. Written informed consent was obtained from all patients participating in the study.

## Results

### Observational study of the current post-stroke situation (Part A)

**Figure 1:**
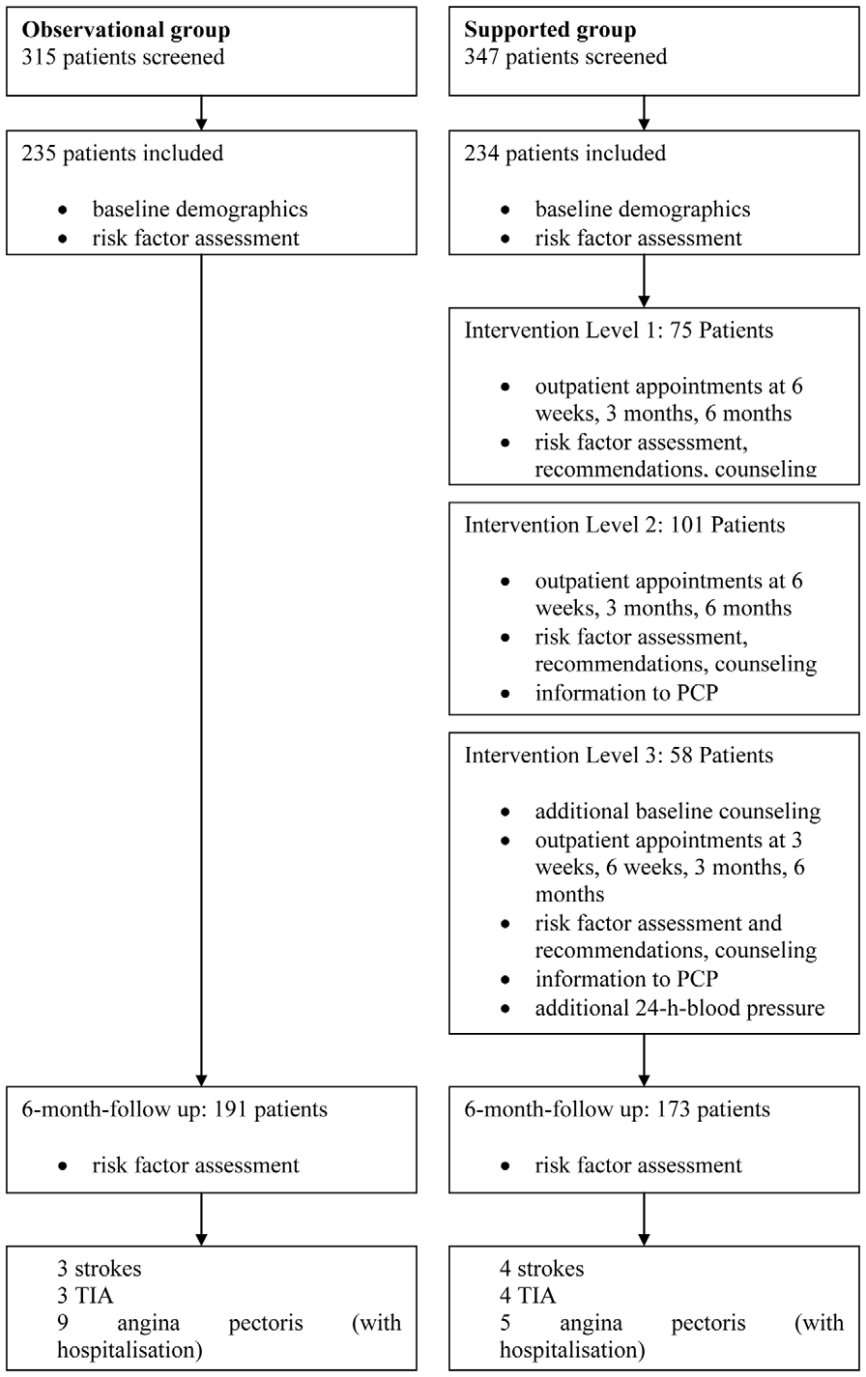
Study flow chart.

From 19^th^ Jan. to 10^th^ Sep. 2009), 303 consecutive patients in the Charité departments of Neurology were screened and 235 patients were included in the study (Figure 1). 191 patients (81.3%) attended the FU visit after 6 months. The prevalence of modifiable risk factors at baseline and at six months is shown in Tables 1 and 2.

**Table 1:** Baseline demographics and risk factors in both cohorts (patients attending 6-month follow-up).

	Observation	Support	p-value
	only*	program	
N	168	173	
Age (years); mean±SD	64.7±11	67.6±10	0.02
TIA** as index event; n (%)	44 (26)	58 (34)	0.21
TOAST-Classification			0.17
Large vessel disease	12 (7)	15 (9)	
Cardioembolic	18 (11)	26 (15)	
Small vessel disease	39 (41)	24 (14)	
Other etiologies	5 (3)	3 (2)	
Not classified	97 (58)	98 (58)	
Intracerebral hemorrhage	0 (0)	3 (2)	
Female gender; n (%)	62 (37)	64 (37)	0.99
Arterial hypertension; n (%)	112 (67)	115 (67)	0.97
Diabetes; n (%)	31 (19)	40 (23)	0.29
Hyperlipoproteinemia; n (%)	85 (51)	87 (50)	0.96
Atrial fibrillation; n (%)	47 (28)	27 (16)	<0.01
Current smoking; n (%)	50 (30)	33 (19)	0.02
Underweight (BMI≤18.5); n (%)	4 (2)	2 (2)	
Overweight (BMI 26–30); n (%)	70 (42)	74 (46)	1.0
Obesity (BMI>30); n (%)	26 (16)	29 (17)	
Physical activity <2 times per week; n (%)	89 (53)	76 (44)	0.10
Admission syst. blood pressure, mmHg	156±26	161±27	0.15
Admission diast. blood pressure; mmHg	84±14	84±14	0.90

* observational patients willing to participate in a future support program.

** without DWI lesion in MRI.

**Table 2:** Baseline demographics and risk factors of observational cohort (patients attending 6-month follow-up, A: willing to participate in a support program, B: not willing to participate in a support program).

	Observation only	Observation only
	A	B
N	168	22
Age (years); mean±SD	64.7±11	67.5±12
TIA* as index event; n (%)	44 (26)	5 (23)
Female gender; n (%)	62 (37)	9 (41)
Arterial hypertension; n (%)	112 (67)	13 (59)
Diabetes; n (%)	31 (19)	1 (5)
Hyperlipoproteinemia; n (%)	85 (51)	11 (50)
Atrial fibrillation; n (%)	47 (28)	7 (32)
Current smoking; n (%)	50 (30)	8 (36)
Underweight (BMI≤18.5); n (%)	4 (2)	0 (0)
Overweight (BMI 26–30); n (%)	70 (42)	6 (27)
Obesity (BMI>30); n (%)	26 (16)	1 (5)
Physical activity <2 times per week; n (%)	89 (53)	6 (27)
Admission syst. blood pressure; mmHg	156±26	151±22
Admission diast. blood pressure; mmHg	84±14	84±14

* without DWI lesion in MRI.

Seven patients (3.7%) had a recurrent event (3 strokes and 4 TIA). Nine additional patients reported angina pectoris with two of them leading to hospitalization.

Of those who participated in the observational study, 168 (88%) assured that they would probably or definitely participate in a prospective controlled trial testing a secondary prevention support program. Baseline parameters of patients stating or not stating their willingness to participate in a support program are shown in Table 2 of the appendix. In order to avoid a bias between patients with or without motivation for a support program, comparisons with the group receiving supported secondary prevention are restricted to patients of the observational cohort who assured that they would probably participate in a prospective trial.

### Intervention study to develop measures for intensified prevention (Part B)

**Figure 2:**
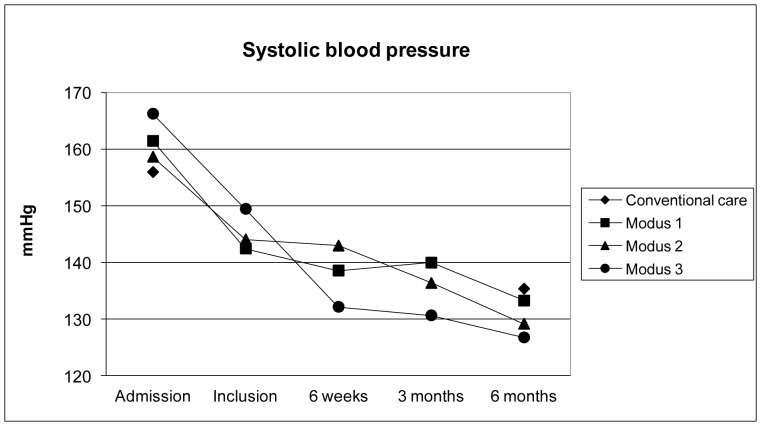
Systolic blood pressure measured during observation period.

From 26^th^ Sept. 2009 to 23^th^ Nov. 2010), 234 consecutive patients were included in the three levels of the support program at the Charité Campus Benjamin Franklin (Figure 2). 78% attended the FU visit after 6 weeks, 79% the FU visit after 3 months and 74% the FU visit after 6-months. 78% attended at least two appointments. After interim evaluations regarding risk factor control and acceptance, supported intervention schemes were modified (level 1: n = 75, level 2: n = 101, level 3: n = 58).

Baseline and follow-up results are shown in Tables 1 and 3 for patients who attended the 6 month appointment. Baseline risk factors were in general similar in the two cohorts except for more frequent atrial fibrillation and more frequently smoking in the observational cohort. Patients of the support program were older and had slightly higher systolic blood pressure at admission.

**Table 3:** 6 month follow-up data (blood pressure values, target values and vascular re-events) in both cohorts.

	Observation only	Support program	p-value
N	168	173	
Syst. blood pressure; mmHg	141±19	130±17	<0.01
Diast. blood pressure; mmHg	83±11	75±9	<0.01
Blood pressure values within recommendations; n (%)	82 (50)	133 (77)	<0.01
LDL<100 mg/dl*; n (%)	88 (63)	117 (71)	0.12
Non-smoking**; n (%)	140 (83)	164 (95)	0.01
AF patients on oral anticoagulation***; n (%)	33 (72)	24 (89)	0,09
INR target (2–3)****; n (%)	17 (42)	17 (56)	0.08
Physical activity according recommendation; n (%)	64 (38)	87 (51)	0.02
Vascular re-event within 6 months; n (%)	7 (4)	8 (5)	0.65

* LDL available in 140 patients of the observational cohort and in 164 patients of the supported cohort.

** In the observational group 25/50 patients (50%) and in the support program 26/33 (79%) quit smoking (p<0.01).

*** Number of patients with AF and documented treatment regarding oral anticoagulation was 54 in the observational cohort and 27 in the support program.

**** Number of patients with AF and INR-measurements at six month was 44 in the observational cohort and 26 in den support program.

**Figure 3:**
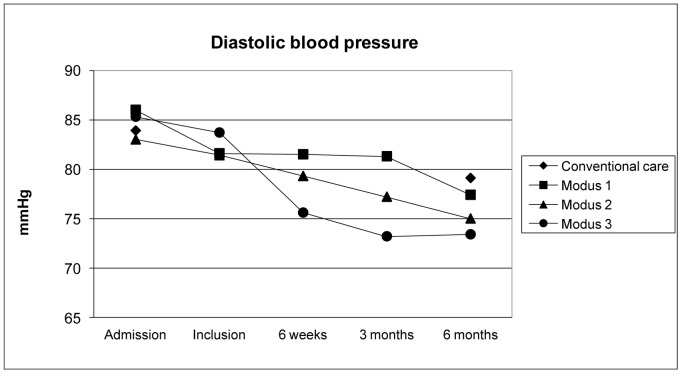
Diastolic blood pressure measured during observation period.

Patients who participated in the support program had a better risk factor control and medical compliance in all assessed parameters. Mean systolic and diastolic blood pressure values over time are shown in Figures 2 and 3. Blood pressure values at 6 months were lower in all levels of the support program compared to the usual care cohort (p<0.01 for both). However, blood pressure lowering was achieved earlier in those who had an appointment already 3 weeks after discharge (level 3). Blood pressure was lowest in the groups with immediate response to elevated blood pressure measured in outpatient appointments (levels 2 and 3).

Eight patients (4.6%) reported a recurrent event (4 strokes and 4 TIA). Five additional patients reported angina pectoris.

## Discussion

This study confirmed that risk factor control after stroke or TIA remained unsatisfactory in our local setting of routine care. Shortly after the index event, patients are obviously motivated to participate in a structured support program. The results after implementation of such a secondary prevention support program suggest that risk factor control can clearly be improved compared to usual care. Intensity of this support relates to the interval until targets are met. However, attendance rates indicate that significant efforts are necessary to keep patients in the program.

Our findings about quality of secondary prevention in usual care are in accordance with previous findings both at a national [24] and international [13], [25], [26] level. A very similar percentage (54.5%) of patients with blood pressure >140/90 mmHg was found at baseline in the Reduction of Atherothrombosis for Continued Health (REACH) Registry [26].

Most studies investigating routine stroke aftercare have reported proportions of medication adherence for different preventive strategies during follow-up so far [13], [27], [28]. Based on the outpatient appointments, we focused on risk factor measurements and achieved target values.

The support program for intensified secondary prevention promises a major reduction of recurrent events when taking into account the high risk for ischemic re-events in acute stroke [29] and TIA patients [9], [30] and the given evidence for risk reduction via blood pressure control [31], LDL-lowering [7] and antithrombotic treatments [3].

The results of our intervention are in accordance with other studies on prevention programs: Supported control of blood pressure was investigated in the Home Blood Pressure study [32] in hypertensive patients. The combination of blood pressure focused website training and regular pharmacist care management including modification of lifestyle yielded superior blood pressure control and improved compliance to pharmaceutical therapy compared to usual care. As a shortcoming of the study, the observation period was one year only and vascular events were therefore not reported as endpoints. Another exemplary randomized study in patients after acute coronary syndrome or stroke showed that risk factors were better controlled under intensive disease management and risk factor modification [33]. A reduction of subsequent vascular events was also suggested within one year.

In this context, it seems of crucial importance that secondary prevention is carried out at an early stage. The EXPRESS study demonstrated successfully that an early and aggressive deployment of combined secondary prevention measures effectively reduces the re-event rate of TIA and minor stroke [11]. It was shown that the 90 days re-event rate both of TIA and minor stroke could be reduced significantly by immediate start of antithrombotic therapy, blood pressure lowering and reduction of cholesterol and, if necessary, initiating carotid endarterectomy. It can be concluded that an improved and early start of secondary prevention is necessary and effective after TIA and minor stroke.

In long-term followed-up patients (mean follow-up 8 years) with type 2 diabetes mellitus, intensive treatment, with a stepwise implementation of behavior modification and pharmacologic therapy that targeted hyperglycemia, hypertension, dyslipidemia, and microalbuminuria, along with secondary prevention of cardiovascular disease with aspirin reduced the risk of cardiovascular and microvascular events by about 50 percent [15].

Our study has several limitations: First, the comparison between usual care and support program was conducted in consecutive but not simultaneous cohorts. We cannot rule out, therefore, that the improved achievement of secondary prevention targets in the support program was at least partially contaminated by an increased awareness about the crucial role of adherence to guidelines both by patients and their family doctors as both studies were performed in the same setting. Second, follow-up data could not be collected in about 20% of patients in both cohorts because we restricted follow-up assessments to those patients who attended the outpatient appointments at six months. Although attendance rates are usually lower than follow-up rates, this mitigates the validity of our results. Third, with the different intervals of outpatient appointments in the two groups, vascular event rates are difficult to compare. Fourth, in contrast to high standard randomized controlled trials, our patient groups, patient characteristics and the way of documentation between both cohorts may differ not only in observed but also in unobserved baseline parameters. The differences in the way of documentation between both cohorts and also in the patient characteristics were not addressed using multivariable analyses. Finally, follow-up was not performed in a blinded manner. In order to minimize selection bias, we only compared patients (usual care cohort) who assured that they would participate in a secondary prevention program with patients in the intervention cohort. Patients who are willing to participate in a supported secondary prevention program are probably more concerned about their health than the general stroke population and more prone to adhere to vascular risk control recommendation.

### Conclusion

The results of our study suggest that the support program leads to a major improvement of secondary prevention. We are going to investigate the effects of this support program on reduction of recurrent coronary and cerebrovascular events in an appropriately powered randomized controlled trial (clinicaltrials.gov: NCT01586702).
